# Mapping unconventional *Leishmania* in human and animal leishmaniasis: A scoping review protocol on pathogen diversity, geographic distribution and knowledge gaps

**DOI:** 10.1371/journal.pone.0332874

**Published:** 2025-09-22

**Authors:** Denis Sereno, Tahar Kernif, Renato Leon, Kholoud Kahime, Souad Guernaoui, Chaymaa Harkat, Mario J. Grijalva, Omar Hamarsheh, Anita G. Villacis, Bachir Medrouh, Thiago Vasconcelos Dos Santos, Razika Beniklef, Naouel Eddaikra, Phlippe Holzmuller

**Affiliations:** 1 UMR177 Intertryp, Institut de Recherche pour le Développement, CIRAD, University of Montpellier, Montpellier, France; 2 GoInsEct: Global Infectiology and Entomology Research Group, France; 3 Laboratory of Parasitic Eco-Epidemiology and Population Genetics, Pasteur Institute of Algeria, Dely-Brahim, Algiers, Algeria; 4 Medical Entomology and Tropical Medicine Laboratory LEMMT, School of Biological and Environmental Sciences, Universidad San Francisco de Quito, Quito, Ecuador; 5 SAEDD Laboratory, School of Technology Essaouira, Cadi Ayyad University of Marrakesh, Morocco; 6 Biotechnology, Conservation and Valorisation of Natural Resources laboratory, Faculty of Sciences Dhar El Mahraz, Sidi Mohamed Ben Abdellah University, Fez, Morocco; 7 Infectious and Tropical Disease Institute, Biomedical Sciences Department, Heritage College of Osteopathic Medicine, Athens, Ohio, United States of America; 8 Department of Life Sciences, College of Science & Technology, Al-Quds University, Jerusalem, Palestine; 9 Centro de Investigación para la Salud en América Latina, Facultad de Ciencias Exactas, Naturales y Ambientales, Pontificia Universidad Católica del Ecuador, Quito, Ecuador; 10 Research Centre for Agropastoralism, Djelfa, Algeria; 11 Parasitology Department, Instituto Evandro Chagas, Ministry of Health, Ananindeua, Brazil; 12 UMR ASTRE, CIRAD, INRAE, University of Montpellier (I-MUSE), Montpellier, France; Universidade Federal da Bahia, BRAZIL

## Abstract

**Introduction:**

Leishmaniases are a vector-borne parasitic diseases with diverse clinical manifestations involving multiple *Leishmania* species and animal hosts. While most leishmaniasis cases are caused by a few well characterized *Leishmania* species, reports describe infections by unconventional or emerging *Leishmania* taxa, atypical clinical presentations from classical species, and occurrences of atypical *Leishmania* in animal hosts. These underrecognized infections present diagnostic and therapeutic challenges and are rarely reflected in surveillance systems or clinical guidelines. A systematic mapping of this evolving landscape is needed to guide future diagnostics, policy, and research priorities.

**Methods and analysis:**

Following the Joanna Briggs Institute (JBI) methodology and PRISMA-ScR guidelines, we will search PubMed, Embase, Cochrane Library (CENTRAL), PROSPERO, Web of Science, and Global Index Medicus, as well as relevant grey literature. Eligible studies will include human cases with clinical presentations that diverge from those typically associated with well-characterized *Leishmania* species, reports involving unconventional or emerging *Leishmania* species, and animal cases of veterinary relevance caused by non-classical species, regardless of study design. Dual independent screening of records and data extraction using a standardized charting form will be conducted. Discrepancies between reviewers will be resolved by consensus. Data will be summarized descriptively through tables, figures, and thematic synthesis. Research gaps will be identified to inform future studies and public health strategies.

**Dissemination:**

This review will use data from published sources and findings will be disseminated through publication in a peer-reviewed journal, presentations at scientific conferences, and sharing with relevant stakeholders. The results are intended to inform clinicians, researchers, and policymakers about the evolving landscape of leishmaniasis and to highlight priorities for future research and surveillance.

## Introduction

### Background

Leishmaniases are vector-borne diseases of significant public health concern, affecting millions of people across the tropics and subtropics (https://www.cdc.gov/dpdx/leishmaniasis/index.html). They present a wide spectrum of clinical manifestations, from cutaneous, diffuse cutaneous and mucocutaneous forms to the potentially fatal visceral leishmaniasis (VL). A vast majority of cases are linked to a limited number of *Leishmania* species. Traditionally, distinct *Leishmania* species have been linked to specific clinical syndromes: *L. donovani* and *L. infantum* are most often associated with VL, while *L. major*, *L. tropica*, and *L. mexicana* are typical causes of cutaneous leishmaniasis (CL) [1]. However, advances in molecular diagnostics have brought attention to a broader spectrum of disease presentations and causative agents. Increasingly, reports describe unconventional species, taxa not historically associated with human infection or that fall outside the small group of well-established species. Some of these are newly characterized or geographically restricted, such as members of the *Mundinia* subgenus, *L. martiniquensis* or *L. orientalis* for example [2,3]. In parallel, a growing number of cases involve non-classical species, including organisms generally considered classical but behaving unexpectedly. Mucocutaneous leishmaniasis (MCL) is a severe, destructive condition primarily caused by *L. braziliensis*, which affects mucous membranes of the nose, mouth, and throat, nevetheless *L. infantum* presenting with mucocutaneous (MCL) forms in North Africa have been described [4]. Diffuse cutaneous leishmaniasis (DCL), is characterized by widespread non-ulcerative skin lesions, associated with an anergic immune response, is most commonly linked to *L. amazonensis or L. aethiopica,* but can be caused by other taxa in immunocompromised individuals [1,5]. These cases challenge existing diagnostic categories and reflect a more complex biological and epidemiological landscape. Finally, infections involve atypical presentations, in which clinical features deviate from established syndromes. This may include unusual disease manifestations, infections in non-traditional hosts (e.g., domestic cats, wild carnivores). Together, these forms, whether involving unusual species, unexpected hosts, or non-canonical symptoms, highlight the need for updated frameworks for diagnosis, surveillance, and treatment.

### Rationale

No global synthesis currently exists to map the diversity, geographic distribution, diagnostic features, and knowledge gaps related to unconventional clinical presentations, atypical *Leishmania* species, and emerging pathogens affecting animals of veterinary interest. These infections often present diagnostic and therapeutic challenges and may be underrecognized within routine surveillance systems [1–3,5,6]. Despite documentation in the scientific literature, these complexities remain underrepresented in the guidance of major stakeholders such as the World Health Organization (WHO), the Centers for Disease Control and Prevention (CDC), and regional health bodies. For example, reports from the European Centre for Disease Prevention and Control (ECDC) and the Pan American Health Organization (PAHO) highlight the lack of standardized diagnostic and treatment protocols across many settings [7–9]. Furthermore, molecular diagnostic methods such as PCR are not widely available in resource-limited regions, posing challenges for the detection and management of infections caused by non-classical *Leishmania* species. This disconnect between emerging evidence and formal guidance underscores the need for a comprehensive synthesis to inform evidence-based policy, strengthen diagnostic and surveillance frameworks, and guide future research priorities. The non-mandatory nature of CL reporting in many endemic countries likely contributes to the underrecognition of atypical presentations and infections involving unconventional or non-classical *Leishmania* species. Without compulsory notification and species-level diagnostics, such cases may go undocumented, obscuring the true diversity and distribution of leishmaniasis and limiting the visibility of emerging epidemiological patterns. Additionally, reports of leishmaniasis emerging in non-endemic regions, whether as imported cases or potential instances of local transmission, further highlight the evolving epidemiological landscape. Such cases often involve atypical clinical forms, unfamiliar *Leishmania* species, or novel reservoir hosts and may be underrecognized due to limited diagnostic capacity and clinical awareness outside traditional endemic zones. These infections are of particular importance for surveillance systems, clinical preparedness, and risk assessment in regions not historically considered at risk. Given the dynamic nature of leishmaniases transmission and the evolving understanding of its causative agents, a scoping review is warranted to compile existing evidence. By identifying and characterizing reports of unconventional *Leishmania* infections in humans and in animals of veterinary relevance, this review will provide a foundational resource for researchers, clinicians, and policymakers. Such mapping is especially timely given the emergence of novel or hybrid strains, shifting ecological patterns, and the increasing application of genomic tools, which have revealed hidden diversity within the *Leishmania* genus [10,11]. A comprehensive overview will help clarify the spectrum of pathogens involved, identify regions at risk, and address the diagnostic and surveillance limitations that currently hinder effective public health responses.

### Objective

The objective of this scoping review is to examine the body of peer-reviewed literature and publications from governmental or non-governmental international sources on unconventional presentations, and pathogens associated with *Leishmania* infections in humans and animals of veterinary importance. This includes infections caused by novel or non-classical *Leishmania* species, atypical clinical manifestations caused by well-known species, and unconventional infections in animal hosts not traditionally associated with the disease. The aim is to define the diversity and global distribution of these occurrences, describe associated clinical and diagnostic patterns, and identify current knowledge gaps. This will be achieved through a multiphase screening and data extraction process. The study aims to answer one overarching research question and several secondary questions.

What is the current global evidence on infections involving unconventional *Leishmania* species, atypical clinical manifestations, or non-traditional *Leishmania* infections in reservoir hosts of medical or veterinary importance and others, with regard to pathogen diversity, geographic distribution, and diagnostic and clinical characteristics?

- What *Leishmania* species, novel, hybrid, or rarely implicated, have been reported in human and animal cases of visceral leishmaniasis (VL), cutaneous leishmaniasis (CL), mucocutaneous leishmaniasis (MCL), diffuse cutaneous leishmaniasis (DCL), or post-kala-azar dermal leishmaniasis (PKDL)?
- What unconventional or atypical clinical manifestations have been associated with either classical or non-classical *Leishmania* species?
- Which animal reservoir hosts, particularly of medical or veterinary importance, have been identified as harboring non-classical or atypically behaving *Leishmania* species?
- In which geographic regions have atypical *Leishmania* infections been reported, and what patterns in spatial distribution can be observed?
- What diagnostic techniques or molecular methods have been used to detect and identify these atypical *Leishmania* infections in both clinical or veterinary contexts?
- What evidence exists in the literature regarding genetic diversity, hybridization events, or adaptative traits among the implicated *Leishmania* strains?
- What treatment strategies have been documented for managing infections caused by non-classical *Leishmania* species or atypical clinical manifestations, and what is known about their outcome or efficacy?
- How have environmental or ecological factors (e.g., climate change, land use changes, urbanization) have been associated with the occurence or detection of atypical *Leishmania* infections?
- What integrated surveillance systems or One Health strategies have been implemented or proposed to monitor and control infections involving unconventional *Leishmania* species?
- What research, diagnostic, or surveillance gaps hinder the detection, understanding, or control of non-classical *Leishmania* infections?

## Methods

This protocol was developed with reference to the JBI Scoping Review Methodology Group’s guidance on conducting scoping reviews and the Preferred Reporting Items for Systematic Reviews and Meta-Analyses, Extension for Scoping Reviews (PRISMA-ScR) reporting guidelines [12,13] (online S1 File). We anticipate data collection will be completed by 30 November 2025, and that data extraction and analysis will begin in December 2025.

### Inclusion and exclusion criteria

For clarity and consistency, we defined the core concepts guiding our inclusion criteria in Table 1. These definitions helped ensure standardized screening and classification of cases across studies.

**Table 1:** Operational definitions of key terms related to unconventional and atypical *Leishmania* infections.

Term	Operational definition	Examples
**Unconventional species**	*Leishmania* taxa that are newly described, geographically restricted, rarely reported in human or animal infection, or not typically included in WHO core species.	*L. martiniquensis*, *L. orientalis*, *L. enriettii*, species in the *Mundinia* subgenus
**Non-classical presentation**	Infection by a well-established *Leishmania* species causing clinical manifestations not typically associated with that species.	Mucosal leishmaniasis from *L. infantum*; visceral leishmaniasis from *L. tropica*
**Atypical infection**	Any case (human or animal) involving an unusual clinical form, anatomical location, reservoir host, or non-endemic geographic setting, regardless of species involved.	Leishmaniasis in cats or wildlife; imported CL cases in non-endemic Europe; disseminated CL

Inclusion Criteria:

- Studies involving human or animal cases (domestic or wild) of leishmaniasis caused by unconventional *Leishmania* species, including newly described, geographically restricted, or rarely implicated taxa (e.g., *L. martiniquensis*, *L. orientalis*, *Mundinia* species).
- Studies reporting atypical clinical presentations or non-classical infections, such as: Unusual anatomical or clinical forms (e.g., mucosal involvement by *L. infantum*, disseminated cutaneous disease), Infections in non-traditional hosts (e.g., cats, rodents, wildlife), infections occurring in non-endemic regions or described as imported.
- All study designs (case reports, case series, observational studies, surveillance studies, clinical trials).
- Studies with species-level identification using molecular, isoenzymatic, or parasitological confirmation (e.g., PCR, DNA sequencing, culture, isoenzyme typing).
- No restriction in language, non-English studies will be translated when feasible.

Exclusion Criteria:

- Studies without species-level identification.
- Reviews, editorials, or commentaries.
- Studies focused solely on classical species (e.g., *L. donovani* in VL, *L. major* in CL).

### Search strategies

Articles will be gathered through searches of PubMed, Embase, Web of Science Core Collection, Cochrane Library, Global Index Medicus, and PROSPERO. PROSPERO will be searched to identify any registered or ongoing evidence syntheses related to atypical or unconventional leishmaniasis to avoid duplication and ensure the novelty of this review. A summary of the rationale for database selection is presented in Table 2. This strategy ensures broad thematic and geographical coverage across both clinical and veterinary domains, consistent with the objectives of this scoping review. There will be no publication date restrictions. Grey literature will be searched via Google Scholar and relevant conference proceedings. The search terms used for each database can be found in online S2 File search terms.

**Table 2:** Justification for database selection.

Database	Rationale for inclusion	Expected contribution
**PubMed**	Broad peer-reviewed biomedical literature coverage, including infectious diseases, diagnostics, and clinical case reports.	Core biomedical studies on *Leishmania*, including human and animal cases and clinical presentations.
**Embase**	Indexes biomedical literature with strong European and global coverage; includes conference abstracts and drug-related studies.	Additional case reports, epidemiological data, and veterinary studies not indexed in PubMed.
**Web of Science Core Collection**	Multidisciplinary database capturing global literature across sciences, including ecology, entomology, and zoonotic diseases.	Non-clinical studies, emerging research, and regionally indexed material on unconventional *Leishmania*.
**Cochrane Library**	Repository of clinical trials and evidence syntheses, including rare disease data and intervention studies.	Relevant trials or systematic reviews on atypical or emerging *Leishmania* forms and treatments.
**Global Index Medicus**	Aggregates literature from WHO regional databases, with emphasis on low- and middle-income countries (LMICs).	Access to local surveillance data and case reports from underrepresented or high-burden regions.
**PROSPERO**	Registry of ongoing and completed systematic reviews.	Identify any registered or ongoing evidence syntheses related to atypical or unconventional leishmaniasis Ensures novelty of the review and avoids duplication of similar protocols.
**Google Scholar**	Collect conference papers, theses, and institutional reports not indexed in standard databases.	Supplementary case reports or surveillance data not formally published but relevant to emerging patterns.

### Record management

Deduplication of abstracts using the De-duplicator from Bond University’s Systematic Review Accelerator will be used [14]. Records will be screened using Rayyan, an article screening tool for evidence synthesis projects [15].

### Screening strategy

The screening process will be conducted in two stages. In the first stage (title and abstract screening), records must meet the primary inclusion criteria and at least one secondary criterion to proceed. This broader approach is intended to avoid the premature exclusion of studies that may not explicitly reference unconventional *Leishmania* infections in the title or abstract but are still relevant. In the second stage (full-text screening), all predefined inclusion criteria must be fully met for a study to be included in the review. All records will be screened independently and in duplicate by two reviewers (excluding MJG). Any discrepancies will be resolved through consensus by a third reviewer (DS or TK). The study selection process will be documented using a PRISMA flow diagram.

### Data extraction

Data extracted from the included texts will follow a standardized, piloted charting form (Table 3). Variables include bibliographic details, study design, clinical and diagnostic features, host species, and outcomes. We will also extract information on the funding source, to assess potential publication or sponsor-related bias and identify patterns in research investment related to non-classical or emerging *Leishmania*. Similarly, surveillance source (e.g., hospital reports, case registries, or active surveillance) will be recorded to contextualize case detection pathways and interpret the representativeness of reported findings.

**Table 3:** Standardized, piloted charting form.

Category	Data	Description/ Notes
**Bibliographic information**	Title	Full title of the article
	Authors	List of authors
	Year of publication	YYYY
	Journal	Name of the publishing journal
	Language	Language of publication
	Funding source	As stated in the article or “Not reported”
**Study characteristics**	Study design	Case report, case series, cross-sectional, surveillance, etc.
	Study setting	Country and region; rural/ urban/ peri-urban if available
	Surveillance source	Passive, active, outbreak investigation, registry, opportunistic, etc.
**Host information**	Host Type	Human, animal (specify: dog, cat, rodent, etc.), both
	Number of cases	Number of unique infections described
	Age/Sex	Aggregated demographic information if applicable
	Immunocompromised host	Yes/ No/ Not reported
**Clinical & Epidemiological details**	Leishmaniasis Type	VL, CL, MCL, DCL, PKDL, Disseminated, Mixed
	Atypical presentation	Yes/No and type (e.g., mucosal from *L. infantum*,)
	Travel or imported case	Yes/ No/ Not reported
	Zoonotic context	Yes/ No – Evidence of animal-to-human link, if any
**Diagnostic methods**	Diagnostic method	PCR, sequencing, culture, microscopy, isoenzyme, RFLP, etc.
	Sample type	Skin biopsy, blood, lymph node, spleen aspirate, etc.
	Diagnostic setting	Point-of-care/ Hospital lab/ Reference lab/ Research only
	Molecular target	ITS1, kDNA, hsp70, SSU rRNA, other (specify)
**Parasitological & Genetic data**	Species identified	Full species name (e.g., *L. martiniquensis*)
	Subgenus	e.g *Viannia*, *Leishmania*, *Mundinia*.
	Hybrid confirmed	Yes/ No/ Suspected
	Parental Species (if hybrid)	As reported (e.g., *L. infantum × L. major*)
	Genetic method	Sequencing, isoenzyme typing, multilocus typing, SNPs
	GenBank accession	If sequence submitted (accession number)
**Vector & Reservoir**	Reservoir host	Species identified as reservoir (e.g., dog, rodent, wildlife)
	Vector species	Species identified as vector
**Treatment & Outcome**	Treatment Given	Drugs, dose, regimen (e.g., miltefosine, amphotericin B)
	Treatment Outcome	Recovered, relapse, died, ongoing, not reported
**Environmental & surveillance factors**	Suspected or proven environmental drivers	Climate change, deforestation, urbanization, land use, etc.
	One health approach described?	Yes/ No (Integration of human, animal, environmental data)
**Knowledge gaps & Author observations**	Reported limitations	As described by authors
	Research gaps identified	Diagnostic, surveillance, taxonomy, etc.
**Reviewer notes**	Comments	Notes or uncertainties

### Data summarization and presentation

Given the anticipated heterogeneity of included studies, spanning diverse host species (humans, domestic animals, others), study designs (case reports to surveillance studies), and diagnostic methods, we will adopt a structured approach to organize and synthesize data thematically and descriptively:

- Stratification by host type (e.g., human, canine, feline, others) to distinguish patterns by reservoir relevance.
- Categorization by clinical form (e.g., CL, VL, MCL, DCL, PKDL, disseminated or mixed presentations).
- Separation by diagnostic approach, noting molecular vs. parasitological confirmation and level of species identification.
- Grouping by geographical region, with attention to endemic vs. non-endemic settings.
- Study design labeling to contextualize the strength and type of evidence (e.g., single case vs. cross-sectional survey).

We will not conduct a formal quality appraisal, consistent with JBI guidance for scoping reviews. However, we will highlight diagnostic certainty and species identification method in the charting form to inform interpretation. We acknowledge potential limitations, including publication bias, inconsistent reporting standards, and challenges in species identification across studies.

### Ethics and dissemination

This scoping review will use data extracted exclusively from publicly available sources, including peer-reviewed publications, surveillance reports, and grey literature such as theses, conference proceedings, and institutional reports. No individual-level or confidential data will be accessed, and no identifiable patient or animal data will be collected. Therefore, formal ethical approval is not required (S3 File).

We acknowledge that grey literature and informal surveillance data may vary in methodological rigor and completeness. To address this, we will extract and report data origin and surveillance type (e.g., passive hospital report, national registry, or academic thesis) and note diagnostic certainty and reporting context when interpreting the findings. These elements will be considered during data synthesis to ensure transparency and contextual clarity.

Findings will be disseminated through publication in a peer-reviewed, open-access journal, presentation at scientific conferences, and sharing with stakeholders in public health, tropical medicine, and veterinary networks.

### Patient and public involvement

There is no applicable patient and public involvement in the study.

### Ethic statement

Patient consent for publication; Not applicable

## Discussion

The goal of this scoping review is to identify and map the global literature on atypical presentations of leishmaniasis and infections caused by unconventional *Leishmania* species. By systematically mapping the available evidence, this review will provide a comprehensive overview of pathogen diversity, geographic distribution, atypical clinical presentations, and diagnostic approaches associated with both unconventional *Leishmania* species and atypical infections, whether in humans or animals of veterinary importance, including those caused by classical species. Given the expanding use of molecular diagnostics and the recognition of atypical presentations, it is likely that the true diversity of *Leishmania* species capable of causing human disease is underestimated. Reports of non-classical species, such as those from the subgenus *Mundinia* or other emerging taxa, highlight the dynamic nature of leishmaniasis epidemiology. This review also captures infections reported in non-endemic or historically low-prevalence regions, which are increasingly relevant du to global travel, ecological shifts, and vector expansion. These cases not only challenge clinician awareness and diagnostic capacity but also underscore the potential for changing transmission patterns. These findings underscore the importance of ongoing surveillance and research, particularly in regions experiencing ecological or demographic changes that may facilitate the emergence of new transmission cycles.

By compiling and synthesizing data from diverse study designs and geographic settings, this review aims to identify not only which unconventional *Leishmania* species are implicated in human and animal infections, but also to characterize atypical clinical presentations and diagnostic approaches, whether associated with emerging species or unusual manifestations of classical pathogens in humans or animals of veterinary relevance. This information is crucial for clinicians and public health practitioners, who may face diagnostic uncertainty when confronted with atypical cases, especially in non-endemic areas or among immunocompromised patients, as well as for public health authorities involved in designing effective surveillance and control programs

Furthermore, mapping research gaps will help guide future investigations. Areas likely to require further study include the clinical spectrum and outcomes associated with infection by unconventional species, the effectiveness of current diagnostic tools, and the potential for misidentification or underreporting in routine surveillance systems. Understanding these gaps will also inform the development of more effective diagnostic algorithms, treatment protocols, and public health strategies.

Ultimately, this scoping review will provides valuable guidance for researchers, clinicians, and policymakers, supporting more accurate diagnosis, reporting, and management of leishmaniasis caused by unconventional species. By highlighting both what is known and what is still uncertain, the review will contribute to a more nuanced and comprehensive understanding of the global leishmaniasis landscape.

### Strenghten and limitations of this study

This is the first scoping review to systematically map unconventional *Leishmania* infections across humans and animals, integrating emerging species, atypical clinical forms, and veterinary hosts within a One Health framework.

The protocol adopts a One Health perspective, enabling integrated analysis of human and animal cases to reflect the complex ecology of *Leishmania* transmission.

Challenges such as publication bias and inconsistent species-level identification may affect data comparability; to mitigate this, we include grey literature, apply broad inclusion criteria, and transparently report data limitations during synthesis.

## Supporting information

S1 File PRISMA-P Checklist. Completed PRISMA-P checklist for reporting systematic review protocols. (DOCX)

S2 File Search Terms. Detailed list of search strategies and terms used in the literature review. (DOCX)

S3 File PLOS One Human Subjects Research Checklist. Completed checklist confirming compliance with human subjects research requirements. (DOCX)
