# Supplementary material for: Mapping unconventional Leishmania in human and animal leishmaniasis: A scoping review protocol on pathogen diversity, geographic distribution and knowledge gaps
Source: PLoS One. 2025 Sep 22;20(9):e0332874. doi: 10.1371/journal.pone.0332874 (PMC12453221; doi:10.1371/journal.pone.0332874)
Supplement: S2 File — (DOCX) [file pone.0332874.s002.docx]

# Supplementary 2 search terms

# **1. MEDLINE (via PubMed)**

# (

# "Leishmaniasis"[Mesh] OR leishmaniasis[tiab]

# OR "cutaneous leishmaniasis"[tiab] OR "visceral leishmaniasis"[tiab]

# OR "mucocutaneous leishmaniasis"[tiab] OR "diffuse cutaneous leishmaniasis"[tiab]

# OR "disseminated leishmaniasis"[tiab] OR "post-kala-azar dermal leishmaniasis"[tiab]

# OR "non-endemic leishmaniasis"[tiab] OR "imported leishmaniasis"[tiab]

# OR CL[tiab] OR VL[tiab] OR MCL[tiab] OR DCL[tiab] OR PKDL[tiab]

# )

# AND

# (

# "Leishmania"[Mesh] OR Leishmania[tiab]

# OR ("non-classical"[tiab] OR "unconventional"[tiab] OR "emerging"[tiab] OR "atypical"[tiab] OR "novel"[tiab]) AND Leishmania[tiab]

# OR "new Leishmania species"[tiab]

# OR "Leishmania (Mundinia)"[tiab] OR Mundinia[tiab]

# OR "L. martiniquensis"[tiab] OR "L. orientalis"[tiab]

# OR (Leishmania[tiab] AND hybrid*[tiab])

# )

# AND

# (

# "Humans"[Mesh] OR humans[tiab]

# OR animals[tiab] OR "reservoir host"[tiab] OR reservoir*[tiab]

# OR dogs[tiab] OR canines[tiab] OR cats[tiab] OR felines[tiab]

# OR horses[tiab] OR wildlife[tiab] OR veterinary[tiab] OR zoonotic[tiab]

# )

# AND

# (

# "molecular diagnosis"[tiab] OR "molecular detection"[tiab]

# OR PCR[tiab] OR "polymerase chain reaction"[tiab]

# OR "species identification"[tiab] OR "DNA sequencing"[tiab]

# OR taxonomy[tiab] OR phylogeny[tiab] OR "genetic diversity"[tiab]

# OR hybridization[tiab] OR genomics[tiab]

# OR "clinical presentation"[tiab] OR diagnosis[tiab]

# OR "geographic distribution"[tiab] OR epidemiology[tiab]

# OR "Case Reports"[ptyp] OR "case series"[tiab]

# )

# **2. PROSPERO**(<https://www.crd.york.ac.uk/prospero>).

# (

# "Leishmaniasis"[Mesh] OR leishmaniasis[tiab]

# OR "cutaneous leishmaniasis"[tiab]

# OR "visceral leishmaniasis"[tiab]

# OR "mucocutaneous leishmaniasis"[tiab]

# OR "diffuse cutaneous leishmaniasis"[tiab]

# OR "disseminated leishmaniasis"[tiab]

# OR "post-kala-azar dermal leishmaniasis"[tiab]

# OR "non-endemic leishmaniasis"[tiab]

# OR "imported leishmaniasis"[tiab]

# OR CL[tiab] OR VL[tiab] OR MCL[tiab] OR DCL[tiab] OR PKDL[tiab]

# )

# AND

# (

# "Leishmania"[Mesh] OR Leishmania[tiab]

# OR ("non-classical"[tiab] AND Leishmania[tiab])

# OR ("unconventional"[tiab] AND Leishmania[tiab])

# OR ("emerging"[tiab] AND Leishmania[tiab])

# OR ("atypical"[tiab] AND Leishmania[tiab])

# OR ("novel Leishmania"[tiab]) OR ("new Leishmania species"[tiab])

# OR "Leishmania (Mundinia)"[tiab] OR Mundinia[tiab]

# OR "L. martiniquensis"[tiab] OR "L. orientalis"[tiab]

# OR (Leishmania[tiab] AND hybrid*[tiab])

# )

# AND

# (

# "Humans"[Mesh] OR humans[tiab]

# OR animals[tiab] OR "reservoir host"[tiab]

# OR "animal reservoir"[tiab] OR reservoir*[tiab]

# OR dogs[tiab] OR canines[tiab] OR cats[tiab] OR felines[tiab]

# OR horses[tiab] OR wildlife[tiab] OR veterinary[tiab]

# OR zoonotic[tiab]

# )

# AND

# (

# "molecular diagnosis"[tiab] OR "molecular detection"[tiab]

# OR PCR[tiab] OR "polymerase chain reaction"[tiab]

# OR "species identification"[tiab] OR "DNA sequencing"[tiab]

# OR taxonomy[tiab] OR phylogeny[tiab]

# OR "genetic diversity"[tiab] OR hybridization[tiab] OR genomics[tiab]

# OR "clinical presentation"[tiab] OR diagnosis[tiab]

# OR "geographic distribution"[tiab] OR epidemiology[tiab]

# OR "Case Reports"[ptyp] OR "case series"[tiab]

# )

# **3. Embase**

# ('leishmaniasis'/exp OR leishmaniasis:ti,ab

# OR 'cutaneous leishmaniasis':ti,ab

# OR 'visceral leishmaniasis':ti,ab

# OR 'mucocutaneous leishmaniasis':ti,ab

# OR 'diffuse cutaneous leishmaniasis':ti,ab

# OR 'disseminated leishmaniasis':ti,ab

# OR 'post-kala-azar dermal leishmaniasis':ti,ab

# OR 'non-endemic leishmaniasis':ti,ab

# OR 'imported leishmaniasis':ti,ab

# OR CL:ti,ab OR VL:ti,ab OR MCL:ti,ab OR DCL:ti,ab OR PKDL:ti,ab)

# AND

# ('leishmania'/exp OR leishmania:ti,ab

# OR (leishmania:ti,ab AND (non-classical:ti,ab OR unconventional:ti,ab OR emerging:ti,ab OR atypical:ti,ab OR novel:ti,ab))

# OR 'new leishmania species':ti,ab

# OR 'leishmania (mundinia)':ti,ab OR mundinia:ti,ab

# OR 'l. martiniquensis':ti,ab OR 'l. orientalis':ti,ab

# OR (leishmania:ti,ab AND hybrid*:ti,ab))

# AND

# ('human'/exp OR humans:ti,ab

# OR 'animal'/exp OR animals:ti,ab

# OR 'reservoir host':ti,ab OR 'animal reservoir':ti,ab OR reservoir*:ti,ab

# OR dogs:ti,ab OR canines:ti,ab OR cats:ti,ab OR felines:ti,ab OR horses:ti,ab OR wildlife:ti,ab

# OR veterinary:ti,ab OR zoonotic:ti,ab)

# AND

# ('molecular diagnosis':ti,ab OR 'molecular detection':ti,ab

# OR PCR:ti,ab OR 'polymerase chain reaction':ti,ab

# OR 'species identification':ti,ab OR 'DNA sequencing':ti,ab

# OR taxonomy:ti,ab OR phylogeny:ti,ab

# OR 'genetic diversity':ti,ab OR hybridization:ti,ab OR genomics:ti,ab

# OR 'clinical presentation':ti,ab OR diagnosis:ti,ab

# OR 'geographic distribution':ti,ab OR epidemiology:ti,ab

# OR 'case report'/exp OR 'case report':ti,ab OR 'case series':ti,ab)**4. Cochrane Library**

# (leishmania OR leishmaniasis OR "leishmania infection"

# OR "cutaneous leishmaniasis" OR "visceral leishmaniasis"

# OR "mucocutaneous leishmaniasis" OR "diffuse cutaneous leishmaniasis"

# OR "disseminated leishmaniasis" OR "imported leishmaniasis" OR "non-endemic leishmaniasis")

# AND

# (non-classical OR unconventional OR emerging OR hybrid OR "novel leishmania"

# OR "new leishmania species" OR mundinia OR "l. martiniquensis" OR "l. orientalis"

# OR "clinical presentation")

# AND

# (humans OR human OR animals OR animal OR "reservoir host" OR "animal reservoir"

# OR dogs OR canines OR cats OR felines OR horses OR veterinary OR zoonotic)

# AND

# ("molecular diagnosis" OR "species identification" OR "dna sequencing"

# OR taxonomy OR "genetic diversity" OR hybridization OR epidemiology

# OR "geographic distribution" OR "case report" OR "case series")

# **5. Web of Science**

TS=(

leishmaniasis OR "cutaneous leishmaniasis" OR "visceral leishmaniasis" OR "mucocutaneous leishmaniasis"

OR "diffuse cutaneous leishmaniasis" OR "disseminated leishmaniasis" OR "post-kala-azar dermal leishmaniasis"

OR "non-endemic leishmaniasis" OR "imported leishmaniasis"

OR CL OR VL OR MCL OR DCL OR PKDL

)

AND

TS=(

leishmania OR "non-classical leishmania" OR "unconventional leishmania" OR "emerging leishmania"

OR "atypical leishmania" OR "novel leishmania" OR "new leishmania species"

OR "Leishmania (Mundinia)" OR mundinia OR "L. martiniquensis" OR "L. orientalis"

OR leishmania* AND hybrid*

)

AND

TS=(

humans OR animals OR "reservoir host" OR "animal reservoir" OR reservoir* OR dogs OR canines

OR cats OR felines OR horses OR wildlife OR veterinary OR zoonotic

)

AND

TS=(

"molecular diagnosis" OR "molecular detection" OR PCR OR "polymerase chain reaction"

OR "species identification" OR "DNA sequencing" OR taxonomy OR phylogeny

OR "genetic diversity" OR hybridization OR genomics

OR "clinical presentation" OR diagnosis OR "geographic distribution" OR epidemiology

OR "case report" OR "case series"

)

**7. Google search**

("unconventional Leishmania" OR "novel Leishmania species" OR "hybrid Leishmania")

AND ("molecular diagnosis" OR PCR OR "atypical leishmaniasis")

AND ("animal host" OR reservoir OR zoonotic)

AND ("case report" OR "thesis" OR "conference")

site:.org OR site:.edu OR site:.int OR site:.gov
